# Obtaining a Diagnostic Yield via Scan findings prior to the introduction of SEquencing retrospectivelY (ODYSSEY): a cohort study

**DOI:** 10.1002/uog.70318

**Published:** 2026-08-14

**Authors:** S. Sonner, C. Mckenna, C. Flanagan, S. Mckee, A. Ververi, S. Doyle, S. Ong, A. J. Mcknight, F. Mone

**Affiliations:** ^1^ Centre for Public Health, Queen's University Belfast Belfast UK; ^2^ Northern Ireland Genomic Medicine Centre, Belfast Health and Social Care Trust Belfast UK; ^3^ Department of Genetics for Rare Diseases Papageorgiou General Hospital Thessaloniki Greece; ^4^ Centre for Prenatal Genetics and Genomics, National Maternity Hospital Dublin Ireland; ^5^ Centre for Fetal Medicine, Royal Jubilee Maternity Service, Belfast Health and Social Care Trust Belfast UK

**Keywords:** exome sequencing, fetal anomaly, fetus, genomic, monogenic, next generation sequencing, prenatal

## Abstract

**Objective:**

To determine the retrospective yield of prenatal exome sequencing (PES) by establishing the proportion of children with a postnatal monogenic diagnosis that could have been diagnosed prenatally if PES had been available.

**Methods:**

The study cohort comprised a sample of children in Northern Ireland, born between January 2010 and January 2018 (predating routine availability of PES), who received a monogenic diagnosis postnatally via next generation sequencing as part of either of two UK‐wide studies (the 100 000 Genomes Project (2015–2018) or the Deciphering Developmental Disorders study (2011–2015)). Clinical data were collected retrospectively and correlated with the current UK National Health Service PES protocol, including the phenotypic eligibility criteria for PES and the associated fetal anomalies gene panel. Cases were considered retrospective diagnoses if the fetal phenotype would have been eligible for PES and the diagnostic gene was included on the test panel, meaning prenatal diagnosis in this current era could have been feasible.

**Results:**

Of 101 children, 17.8% (95% CI, 10.3–25.3%) had both an eligible fetal structural anomaly (FSA) (i.e. high‐risk FSA) and a diagnostic gene on the associated test panel, meaning that they could have been diagnosed prenatally in the current clinical landscape. The median length of the diagnostic odyssey for this subgroup of children was 3.7 years (1354 (range, 822–2450) days). Moreover, 58.4% (*n* = 59) of cases had no anomalies detected prenatally and 19.8% (*n* = 20) had a FSA that would not meet the eligibility criteria for PES (low‐risk FSA). Although these cases would have been ineligible for PES under the current clinical pathway, 89.9% (*n* = 71/79) were affected by severe or profound syndromes. Postnatally, the most common functional anomalies were neurodevelopmental delay/intellectual disability and/or behavioral abnormality, which were observed in 80.2% (*n* = 81) of the included children. However, 80.2% (*n* = 65/81) of these affected children did not present with fetal anomalies eligible for PES.

**Conclusions:**

Almost one‐fifth of children with a monogenic condition included in this study could have received a diagnosis via modern PES, avoiding a diagnostic odyssey lasting almost 4 years. However, despite having a monogenic condition, over half of the children did not present with any structural anomalies *in utero*. This demonstrates the degree to which fetal imaging is limited in its ability to reassure parents of the absence of a fetal genetic syndrome. © 2026 The Author(s). *Ultrasound in Obstetrics & Gynecology* published by John Wiley & Sons Ltd on behalf of International Society of Ultrasound in Obstetrics and Gynecology.

## INTRODUCTION

In clinical genetics, the diagnostic odyssey refers to the often prolonged and complex process patients and their families may face in order to obtain an accurate diagnosis of a rare disease. There are > 8000 known rare diseases, making them difficult to diagnose. This can lead to patient anxiety and may limit access to quality‐of‐life‐improving care^1^. Approximately 80% of rare diseases have a monogenic origin, therefore the clinical adoption of next generation sequencing has increased diagnostic yields and reduced turnaround times compared with traditional testing methods^2^.

For some, the diagnostic odyssey can begin in the prenatal period, as 2–3% of pregnancies worldwide are affected by fetal structural anomalies (FSA) that can be detected on ultrasound. Traditional chromosomal testing methods, including karyotyping and chromosomal microarray, explain up to 40% of these anomalies, primarily through the detection of aneuploidy or copy number variants. The remaining inconclusive cases may be the result of pathogenic single gene variation. Establishing a molecular prenatal diagnosis supports decisions regarding pregnancy course and management through to the neonatal period and it can also inform subsequent pregnancies^3^.

Prenatal exome sequencing (PES) following FSA detection has shown a 31% incremental diagnostic yield over traditional genomic testing methods^4^. Within the UK National Health Service (NHS), PES became a commissioned clinical service in October 2020 under the R21 pathway^5^. Eligibility for testing is determined using evidence‐based phenotypic inclusion criteria that identify fetuses with FSA most likely to have an underlying monogenic disorder, with exome data subsequently analysed against a virtual panel of ‘green genes’ with established gene–disease associations.

This study aimed to retrospectively evaluate the clinical utility of PES in a cohort of children with confirmed monogenic disorders from two previous studies who were born before the routine implementation of PES and were therefore diagnosed postnatally. Using modern NHS protocols as a model, the key objectives were to determine: (1) the retrospective yield, i.e. the proportion of cases hypothetically diagnosable via PES; (2) the length of patients' diagnostic odysseys; (3) the incidence and relationships of prenatal and postnatal findings; and (4) the severity of the diagnosed syndromes and their relation to prenatal presentation.

## METHODS

### Cohort selection and data collection

The cohort consisted of children in Northern Ireland (NI) who obtained a molecular diagnosis from whole genome sequencing or whole exome sequencing via local recruitment from a NI‐based hub (Belfast City Hospital, Belfast, UK) to one of two UK‐wide studies: the 100 000 Genomes Project (2015–2018)^6^ or the Deciphering Developmental Disorders study (2011–2015)^7^. The methodologies of both studies have been described previously^8^, ^9^. Clinical services were provided by the NI Regional Clinical Genetics Service (NIRCGS) and laboratory and interpretation services were provided by the NI Regional Molecular Diagnostics Service (NIRMDS). These services are provided under Health and Social Care NI (the NHS body in NI). PES is not internally commissioned by the NIRCGS/NIRMDS but has been accessed as an outsourced service provided by NHS laboratories in England since 2022.

Inclusion criteria for the Obtaining a Diagnostic Yield via Scan findings prior to the introduction of SEquencing retrospectivelY (ODYSSEY) cohort were children with a postnatal monogenic diagnosis with validated causative pathogenic/likely pathogenic variant(s), as per the American College of Medical Genetics and Genomics (ACMG)^10^ and Association for Clinical Genomic Science (ACGS)^11^ guidelines, who were born between January 2010 and January 2018 (before routine availability of PES). Cases were included if they were conceived after the adoption of Viewpoint (GE Healthcare, Zipf, Austria)^12^, an administrative software used to log fetal imaging records.

Ethical approval had been acquired previously for the participants during initial enrolment in their original studies (MREC (04/MRE05/50)). Participants gave consent to allow additional analysis of their clinical data for service development research, and they were given a pseudonymized study ID to protect their identities.

Data were collected retrospectively from electronic care records (Viewpoint, laboratory information management systems and the Decipher database^13^) and were cross‐referenced by two researchers (F.M. and S.S.) for quality assurance.

### Retrospective analysis of ODYSSEY

For a rare disease case to be prenatally diagnosable, three factors had to be fulfilled: anomaly detection, eligibility for genomic testing and gene coverage. The retrospective yield was determined as the proportion of cases that could have been diagnosed via PES, using the current NHS R21 criteria^5^. Retrospective prenatally diagnosable cases were those with a FSA documented in their prenatal records that would have met current NHS eligibility criteria for PES (Figure S1), and whose confirmed disease‐causing gene was included on the associated test panel, which is regularly updated, as of April 2026.

First, prenatal records were searched. Cases in which a FSA was noted were categorized by two fetal medicine specialists (F.M. and S.O.) into three phenotypic subcategories based on the perceived risk of underlying genetic disease and eligibility for PES. The categories were: (1) high‐risk FSA (HR‐FSA) (findings considered high risk for genetic disease that met the NHS PES inclusion criteria); (2) low‐risk FSA (LR‐FSA) (findings considered low risk for genetic disease that did not meet the NHS PES inclusion criteria); and (3) no FSA (no indicators for pursuing further testing).

The diagnostic genes associated with the HR‐FSA cases were searched on the NHS virtual panel (Fetal Anomalies version 6.0)^14^, hosted on PanelApp^15^. Green genes are included on the test panel if there is an established gene–disease relationship (Figure S2). Cases that met phenotypic inclusion criteria and were linked to a green gene were deemed part of the actual retrospective yield. This required the assumption that parents in all eligible cases would have opted for further prenatal testing, which would have required invasive sample collection via amniocentesis or chorionic villous sampling.

The actual retrospective yield only included cases in which a HR‐FSA was noted in the prenatal period. However, some cases showed eligible structural anomalies that could have been detected via prenatal ultrasound but were noted neonatally. Therefore, the fetal medicine specialists also investigated the neonatal records of cases with LR‐FSA and no FSA. If cases had presented on neonatal examination with additional anomalies typically detectable via prenatal ultrasound that would meet phenotypic inclusion criteria for PES, they were considered possible additional HR‐FSAs^16^. The diagnostic genes for these cases were also searched on the virtual panel and a maximum possible yield (the cases included in the actual retrospective yield and the cases with neonatally identified HR‐FSAs and a diagnostic gene included on the virtual panel) was established. This required the further assumption that, hypothetically, these additional relevant neonatal anomalies would have been detected prenatally, triggering referral for PES under the NHS R21 pathway.

### Length of the diagnostic odyssey

The length of the diagnostic odyssey was estimated from the age of the patient at diagnosis (diagnostic report issue) in calendar days. The median odyssey length was found for cases in the overall cohort, the actual retrospective yield cohort and the maximum possible yield cohort.

### Phenotypic presentation and syndrome progression

The incidence of children affected by structural and functional anomalies was established. Pediatric phenotypes were reported as human phenotype ontology^17^ terms provided by the referring clinician on clinical genetics reports.

Diagnosed syndromes were classified by their severity, using criteria published previously^18^, ^19^ and agreed upon by two genetics experts (A.V. and S.D.). This system evaluates features associated with a genetic syndrome based on their impact on life expectancy, cognitive function, mobility, physical malformation and other parameters (Figure S3). Syndrome severity was classified as mild, moderate, severe or profound, based on traits observed in ≥ 25% of the affected population.

### Statistical analysis

The actual retrospective yield and maximum possible yield were calculated as percentages of the total cohort. To account for uncertainty, binomial proportion 95% CIs were calculated for each yield using SPSS version 31.0 (IBM Corp., Armonk, NY, USA). The median length of the diagnostic odyssey was converted to years for digestibility and presented with the range (given in days).

## RESULTS

### Retrospective yield

**Figure 1:**
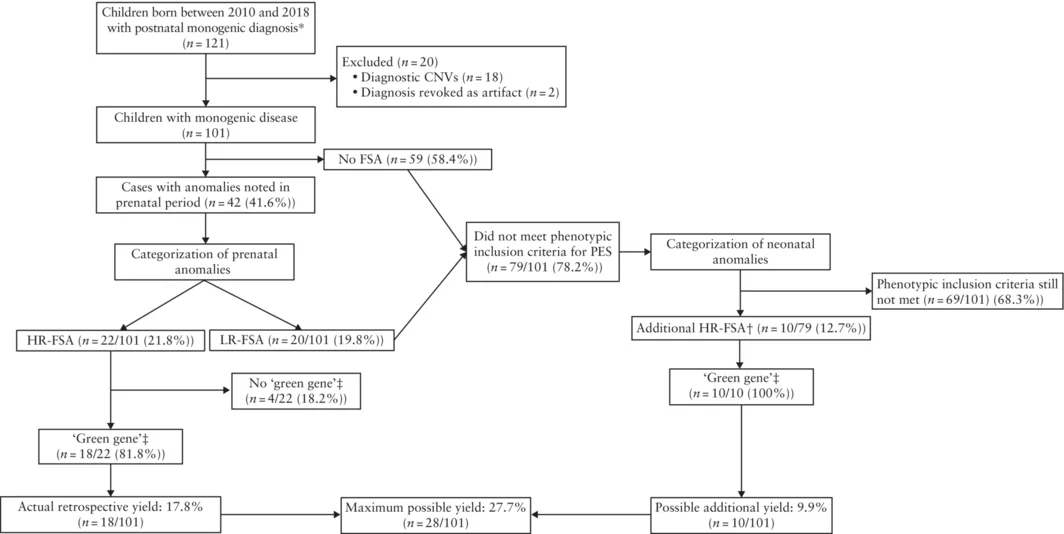
Summary of the Obtaining a Diagnostic Yield via Scan findings prior to the introduction of SEquencing retrospectivelY (ODYSSEY) cohort and steps to estimate retrospective yield of prenatal exome sequencing (PES) in children diagnosed postnatally with monogenic disease, modeled by UK National Health Service (NHS) protocol. *Cases in cohort identified from all rare disease patients recruited via Belfast City Hospital, Belfast, UK, for the 100 000 Genomes Project^6^ and the Deciphering Developmental Disorders study^7^. †Cases with additional anomalies noted in the neonatal period that can typically be detected by prenatal ultrasound and would qualify for PES under NHS selection protocol. ‡‘Green gene’ refers to diagnostic genes included on the NHS test panel for PES, indicating that there is an established gene–disease relationship. CNV, copy number variant; FSA, fetal structural anomaly; HR‐FSA, high‐risk FSA; LR‐FSA, low‐risk FSA.

Overall, 101 children born in NI between 2010 and 2018 were included in the study cohort. Figure 1 breaks down cohort selection and the subsequent steps to determine the retrospective yield. In total, 42 (41.6%) cases had prenatal anomalies detected, of which 22 (21.8%) had a HR‐FSA and therefore would have qualified for PES under current guidance, while 20 (19.8%) cases had a LR‐FSA, and 59 (58.4%) showed no FSAs. Of the HR‐FSA cases, 18/22 (81.8%) had their diagnostic gene included on the NHS virtual fetal panel (green gene), resulting in an actual retrospective yield of 17.8% (95% CI, 10.3–25.3%). Of these, the majority (14/18 (77.8%)) were *de novo* variants and 4/18 (22.2%) were inherited.

The remainder of the cohort had LR‐FSAs or no FSAs (*n* = 79 (78.2%)). However, 10 of these cases had additional anomalies recorded neonatally that are typically detectable on prenatal ultrasound and would have been eligible for PES. All diagnostic genes for these additional cases were found on the virtual panel, resulting in a maximum possible yield of 27.7% (95% CI, 19.0–36.4%) in the cohort. Phenotype–genotype data for all cases are presented in Tables S1–S5.

The median diagnostic odyssey length for the entire cohort was 5.7 years (2081 (range, 723–4562) days). For the 18 patients included in the actual retrospective yield, the median diagnostic odyssey length was 3.7 years (1354 (range, 822–2450) days) and, for the patients included in the maximum possible yield, the median diagnostic odyssey length was 3.8 years (1380 (range, 723–3975) days).

### Pre‐ and postnatally detected anomalies

Within the HR‐FSA cases, the most common inclusion criterion for PES met was complex central nervous system anomalies (*n* = 8/22 (36.4%)), followed by isolated growth restriction and anomalies in multiple systems (*n* = 6/22 (27.3%) cases each). The most common LR‐FSA anomalies, which would not be eligible for PES, were growth restriction with placental insufficiency, isolated facial dysmorphism and amniotic fluid anomalies, all of which were seen in 4/20 (20.0%) cases each.

**Figure 2:**
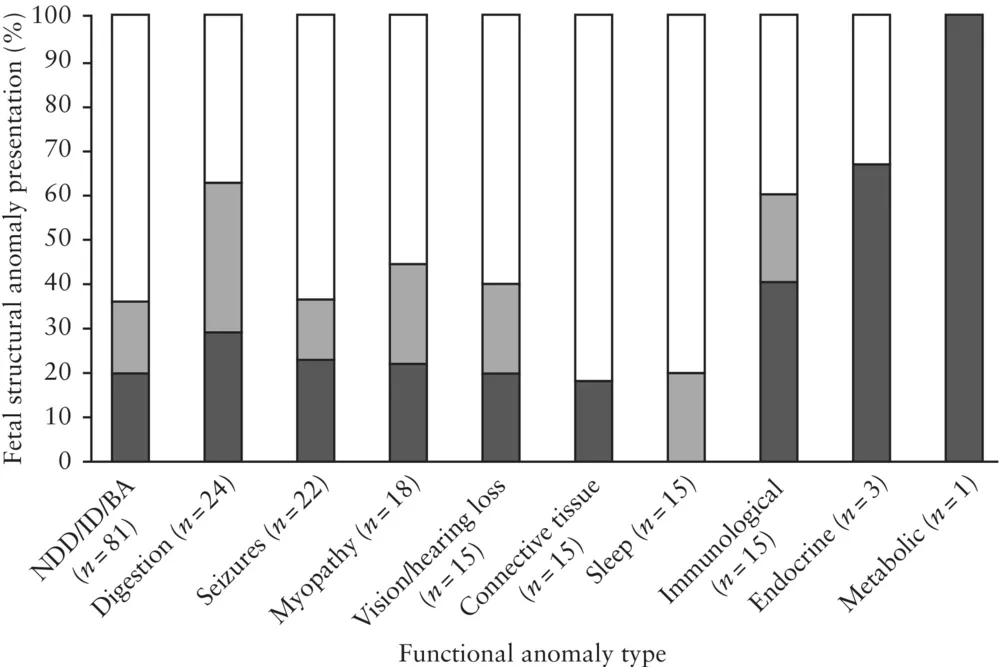
Incidence of functional anomalies, classified into 10 categories, in 101 children with postnatal diagnosis of monogenic disease, according to how affected children presented prenatally: high‐risk fetal structural anomaly (FSA) (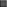), low‐risk FSA (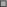) or no FSA (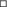). Fifty‐three children were affected by at least two anomaly types. NDD/ID/BA, neurodevelopmental delay/intellectual disability and/or behavioral abnormality.

The proportions of children affected by functional anomalies in 10 categories are shown in Table S5 and the corresponding prenatal presentations of affected children are shown in Figure 2. The most common functional anomalies were neurodevelopmental delay/intellectual disability and/or behavioral abnormality (NDD/ID/BA), which were seen in 81 (80.2%) cases. According to the prenatal records of these affected children, 16/81 (19.8%) presented *in utero* with a HR‐FSA, 13/81 (16.0%) with a LR‐FSA and 52/81 (64.2%) with no FSA.

### Syndrome severity

The severity classifications of the syndromes observed in the cohort are summarized in Table 1. Of all the diagnosed genetic syndromes, two (2.0%) were classified as mild, nine (8.9%) as moderate and 90 (89.1%) as severe or profound. The majority (71/90 (78.9%)) of severe or profound syndromes would not have been eligible for PES based on the current UK selection protocol.

**Table 1:** Incidence of fetal structural anomalies (FSAs) identified in children with monogenic diseases diagnosed postnatally (*n* = 101), according to severity of the disease

Syndrome severity classification	HR‐FSA (*n =* 22)	LR‐FSA (*n =* 20)	No FSA (*n =* 59)	Total (*n =* 101)	Prenatally diagnosable*
Mild	0 (0)	0 (0)	2 (3.4)	2 (2.0)	0/2 (0)
Moderate	3 (13.6)	1 (5.0)	5 (8.5)	9 (8.9)	2/9 (22.2)
Severe or profound	19 (86.4)	19 (95.0)	52 (88.1)	90 (89.1)	16/90 (17.8)

Data are given as *n* (%) or *n*/*N* (%).

* Cases in which findings noted on prenatal records met National Health Service (NHS) phenotypic inclusion criteria for prenatal exome sequencing and the diagnostic gene was included on the associated test panel (actual retrospective yield). HR‐FSA, high‐risk fetal structural anomaly; LR‐FSA, low‐risk fetal structural anomaly.

## DISCUSSION

This retrospective study indicated that modern PES could have diagnosed 17.8% of children diagnosed postnatally with a monogenic disease in a cohort derived from two earlier studies^8^, ^9^. As PES was not available during the gestation of these cases, the children encountered a median diagnostic odyssey of 3.7 years. Notably, 58.4% (59/101) of cases showed no FSAs but 88.1% (52/59) of these cases went on to have profound or severe conditions.

Our findings provide additional evidence supporting the use of PES in the early diagnosis of rare diseases^20^. We have also highlighted gaps in prenatal diagnosis, particularly regarding phenotype‐driven case selection. While using phenotypic criteria and targeted gene panels can optimize resource use and minimize the return of incidental findings and variants of unknown significance (VUS)^21^, diagnoses may be missed. Of eligible cases with FSAs identified prenatally, 18.2% (4/22) had causative genes that were not included on the virtual fetal panel. However, this panel is iterative and updated regularly to reflect newly discovered gene–disease relationships.

Fetal phenotyping is complex. The fetal presentation evolves during gestation and the level of phenotypic information available is limited to the structural anomalies that are identifiable on ultrasound^22^. The postnatal presentation of a genetic syndrome may be known but its fetal presentation may not have been observed previously. FSA detection depends on factors such as scan timing, the sonographer's skill and maternal body composition^23^. NHS PES is initiated following FSA detection, and detection rates can be highly variable. In England, congenital anomalies are detected antenatally in approximately 63% of affected babies^24^. In the current study, we retrospectively identified 10 cases affected by structural anomalies that were only reported in the neonatal period but typically would be prenatally detectable and would be eligible for PES in the current clinical setting, in addition to the 22 cases with HR‐FSAs that were detected at the original prenatal examination. Therefore, the FSA detection rate within this study (68.8% (22/32)) is similar to that reported by the NHS in England. Deep prenatal phenotyping is very important, especially if PES eligibility is phenotype based. With 100% FSA detection, our retrospective yield could increase from 17.8% to 27.7%.

Moreover, the possibility of an underlying monogenic disease is not ruled out for fetuses that are phenotypically ineligible for PES, as no eligible FSA was found on either the prenatal or neonatal records in 68.3% of cases. Some genetic syndromes may be later onset or have mostly functional symptoms that cannot be seen on ultrasound. Over half of the cases with NDD/ID/BA showed no fetal indicators. Similar results have been found in another study in which 52.5% of neurocognitive disorders diagnosed after birth showed no FSA^25^. Generalized phenotypic eligibility criteria have been found to be an inefficient approach to PES case selection. Instead, a comprehensive case‐by‐case analysis where possible is optimal^26^.

Many PES experts are now discussing sequencing ‘phenotypically normal’ fetuses as a possible solution for how monogenic cases may be dismissed for testing owing to their phenotype. Our findings could be interpreted in support of this, as most cases were not associated with FSAs. However, sequencing fetuses without anomalies creates specific ethical and clinical challenges and is not supported by any current international governing body^27^. Uniquely, diagnostic prenatal results can be actionable and irreversible if parents opt to terminate their pregnancy. Clinicians should take a rigorous approach to prenatal diagnosis and subsequent counseling to minimize parental distress. Most of our cohort showed no features of disease until the childhood onset of functional anomalies; had their diagnostic variant been detected prenatally, it may have been classified as a VUS. Although this classification could have been upgraded to pathogenic after birth, it may have caused uncertainty during the pregnancy. This highlights the importance of revisiting inconclusive cases, particularly in the neonatal period when more phenotypic information is accessible, to increase yields without collecting an additional patient sample. Further research is still required to understand the utility of sequencing structurally normal fetuses, and expert consensus is needed to navigate any development of clinical and laboratory guidance.

The main strength of our study is our clinically impactful findings in the current era of prenatal genomics. We demonstrate PES utility in an NI sample, where access has been limited, and investigate factors hindering prenatal diagnosability. Our cohort comprised patients affected by a wide range of syndromes, reflecting the diverse nature of conditions seen within clinical genetics services. We were able to summarize the variable trajectories of these diseases with the severity‐classification model. However, we note the severity system's limited flexibility, and some cases required individual clinical judgment and hence results should be interpreted with caution. To our knowledge, we used the most prevalent and widely accepted classification system available at the time.

The nature of our analysis created some limitations in study design, namely cohort selection bias. All patients had a confirmed genetic diagnosis and had survived to a stage at which postnatal testing was offered. This does not reflect the population typically referred for PES, in which many cases end in intrauterine or neonatal death. Therefore, the findings of our study should be interpreted with caution as they are specific to this postnatally diagnosed cohort. Our primary outcome, retrospective yield, is not a standard clinical diagnostic yield of PES, but rather the proportion of cases with monogenic disease that were prenatally diagnosable. Moreover, the frequencies of syndrome severity and anomaly incidence only represent this highly selected sample, not distributions seen in the normal population.

The analysis relied on retrospective data, which are often limited and/or inconsistent, and it required significant assumptions to be upheld^28^. To control for this, variables were collected and cross‐referenced from up to four sources and validated by two researchers. All key decisions such as phenotypic eligibility and syndrome severity were agreed by two researchers to minimize bias. Additionally, the retrospective diagnoses are upheld by assumptions regarding testing acceptability and anomaly detection, and 95% CIs have been included to account for this uncertainty.

In conclusion, when striving to reduce diagnostic odysseys, genetic investigation in the prenatal period is an important area for focus. All syndromes may not be prenatally diagnosable if genotype and/or phenotype data are lacking. However, long‐term storage of sequencing data allows inconclusive cases to be revisited after birth. Although many syndromes will not have distinctive or clear fetal presentations, further work is necessary before any progress is made on sequencing apparently normal fetuses. Going forward, in clinical practice, parents should be counseled appropriately to manage their expectations of PES and to ensure that they understand the possible prenatal and postnatal outcomes of genomic sequencing.

## Supporting information


**Figure S1** National Health Service (NHS) England R21 pathway inclusion and exclusion criteria of fetal structural anomalies which determine if a pregnancy is eligible for prenatal exome sequencing^1^.
**Figure S2** Classification system applied to genes within virtual panels curated by Genomics England for genetic disease diagnosis^2^, ^3^.
**Figure S3** Flowchart showing algorithm employing published criteria to classify genetic syndromes based on their severity^4^.

**Table S1** Phenotype and genotype information for all prenatally diagnosable cases included in the study, divided into cases included in the actual retrospective yield (cases that could have been identified by prenatal exome sequencing (PES), according to National Health Service (NHS) England R21 pathway, with a diagnostic gene on the associated testing panel (‘green gene’)) (*n* = 18) and additional cases included in the maximum possible yield (cases with neonatally identified anomalies that can typically be detected via prenatal ultrasound scan and would qualify for PES under NHS selection protocol and have a green gene) (*n* = 10).
**Table S2** Phenotype and genotype information for cases that presented with high‐risk fetal structural anomalies according to patient's prenatal records (*n* = 22).
**Table S3** Phenotype and genotype information for cases that presented with low‐risk fetal structural anomalies according to patient's prenatal records (*n* = 20).
**Table S4** Phenotype and genotype information for cases that presented with no fetal structural anomalies according to patient's prenatal records (*n* = 59).
**Table S5** Incidence of functional anomalies and the proportion of affected children who presented prenatally with high‐risk fetal structural anomalies (FSA), low‐risk FSA or no FSA (*n* = 101).

## Data Availability

The data that supports the findings of this study are available in the supplementary material of this article.
