# Supplementary material for: Obtaining a Diagnostic Yield via Scan findings prior to the introduction of SEquencing retrospectivelY (ODYSSEY): a cohort study
Source: Ultrasound Obstet Gynecol. 2026 Aug 14;68(3):388–94. doi: 10.1002/uog.70318 (PMC13532116; doi:10.1002/uog.70318)
Supplement: Supplementary file 1 [file UOG-68-388-s002.docx]

**Supplementary Methods**

a. Phenotypic Inclusion Criteria –

Cases referred to R21 pathway for prenatal exome sequencing (ES) if phenotype observed on ultrasound aligns with the evidence-based inclusion criteria – Figure S1, based on the likelihood of findings being caused by monogenic disease.

Figure S1 - NHS England R21 inclusion and exclusion criteria of fetal structural anomalies which determine if pregnancy is eligible for prenatal exome sequencing.^1^

b. Green Genes –

Virtual gene panels employed by the NHS prioritise variants found within select genes which have a known relationship to the specific phenotype under investigation. Genes are classified within panels according to a traffic light system.

Pathogenic variation within green genes have sufficient evidence of causation of the phenotype. For example, green genes on the R21 virtual panel have a known link to fetal anomalies.

Figure S2: Classification system applied to genes within virtual panels curated by Genomics England for genetic disease diagnosis.^2,3^

c. Classification of Syndrome Severity –

Criteria to classify genetic syndromes into categories based on severity has been previously published. Syndromes classified as profound, severe, moderate and mild.

Figure S3 – Flow chart showing algorithm which employs a published criteria to classify genetic syndromes based on their severity.^4,5^

References

1. NHS East Genomics. National Genomic Test Directory (V5 April 23) Inclusion criteria for rPES (R21) – Fetal Medicine. R21 inclusion criteria and pathway and checklist for clinicians. Available online at <https://www.eastgenomics.nhs.uk/about-us/genomic-medicine-service-alliance/Transformation_Projects/transformation-project-archive/coordination-of-genomic-services-in-prenatal-care-within-the-east-gmsa-region/r21_inclusioncriteria_pathwaychecklist/>

Last accessed 27/10/2025.

2. Sarah Leigh, et al. Genomics England Quality Management System – PanelApp Handbook. GUI-BIO-004. v35. 10/11/2022. Pg 13, 8.5 Understanding Gene Ratings on a Version 1+ Gene Panel.
3. Genomics England. Genomics England Research Environment User Guide. Rare disease tiering. https://re-docs.genomicsengland.co.uk/tiering/#rare-disease-tiering. Accessed 11/03/2025.
4. Arjunan, Aishwarya et al. “Evaluation and classification of severity for 176 genes on an expanded carrier screening panel.” *Prenatal diagnosis* vol. 40,10 (2020): 1246-1257. doi:10.1002/pd.5762
5. Lazarin, Gabriel A et al. “Systematic Classification of Disease Severity for Evaluation of Expanded Carrier Screening Panels.” *PloS one* vol. 9,12 e114391. 10 Dec. 2014, doi:10.1371/journal.pone.0114391
